# Directing and Understanding the Translation of a Single Molecule Dipole

**DOI:** 10.1021/acs.jpclett.2c03472

**Published:** 2023-03-03

**Authors:** Grant J. Simpson, Víctor García-López, A. Daniel Boese, James M. Tour, Leonhard Grill

**Affiliations:** † Department of Physical Chemistry, Institute of Chemistry, University of Graz, Heinrichstrasse 28, 8010 Graz, Austria; ‡ Departments of Chemistry and Materials Science and NanoEngineering and Smalley-Curl Institute and NanoCarbon Center, Rice University, Houston, Texas 77005, United States; § Department of Theoretical Chemistry, Institute of Chemistry, University of Graz, Heinrichstrasse 28, 8010 Graz, Austria

## Abstract

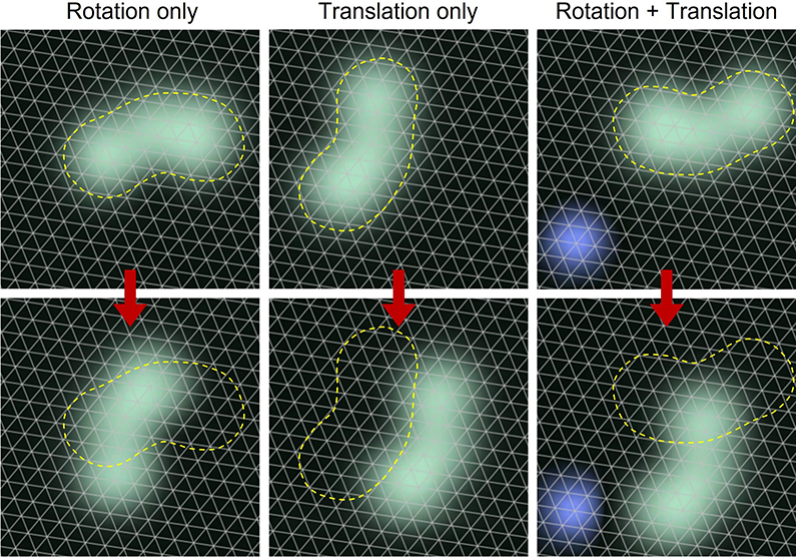

Understanding the directed motion of a single molecule on surfaces is not only important in the well-established field of heterogeneous catalysis but also for the design of artificial nanoarchitectures and molecular machines. Here, we report how the tip of a scanning tunneling microscope (STM) can be used to control the translation direction of a single polar molecule. Through the interaction of the molecular dipole with the electric field of the STM junction, it was found that both translations and rotations of the molecule occur. By considering the location of the tip with respect to the axis of the dipole moment, we can deduce the order in which rotation and translation take place. While the molecule–tip interaction dominates, computational results suggest that the translation is influenced by the surface direction along which the motion takes place.

Understanding how molecules translate over a surface is crucial for understanding many phenomena, including the growth of two-dimensional materials,^1^ catalytic processes,^2^ and on-surface polymerization.^3^ The ability to direct such translations, and hence overcoming the random behavior intrinsic at the small scale, plays a key role in designing molecular machines capable of performing meaningful work based on rotation and/or translation. While not as complex as their biological counterparts,^4^ artificial molecular machines^5^ are diverse in their design and function and include switches,^6^ pumps,^7,8^ and motors.^9,10^ Directed translation can come from an intrinsic property of the molecule that can be triggered, for example, by light^11^ or chemical fuel.^12^ While many cases of molecular machines were observed in solution, a few studies were also performed at surfaces.^13−15^ On the contrary, molecules that do not possess any intrinsic means of directed motion are much more common and can be controlled by external stimuli such as an electric field.^16^ In this regard, the scanning tunneling microscope (STM), in which a strong electric field exists in the junction between its metallic tip and the surface, provides a perfect tool for manipulating molecules at the individual level^17−20^ while delivering subangstrom spatial resolution. STM tip-induced translations are known and have been used to direct the motion of molecules on surfaces during the first international nanocar race.^21^ Indeed, further investigation^22^ and design^23,24^ of molecules with permanent dipole moments are ongoing. In the work presented here, we present such a single molecule dipole and demonstrate how its translation can be not only controlled but also understood.

In this study, 2,5-di(ethynyladamantanyl)-4-(dimethylamino)nitrobenzene (DDNB) molecules^19,20^ were studied on a single-crystal Ag(111) surface. DDNB, shown in Figure 1a, consists of a central phenyl ring with dimethylamine [-N(CH_3_)_2_] and nitro (-NO_2_) groups positioned opposite each other. These groups possess strong electron-donating and -withdrawing properties, respectively, and hence, a permanent dipole moment exists along the short axis of the molecule. The two ethynyladamantanyl groups on either side of this central portion serve to lift the molecule up from the surface and prevent strong interaction of the polar groups with the surface. Calculations^20^ of an isolated molecule in its surface geometry have shown that the molecular dipole moment (in total 8.4 D as compared to 6.7 D in its gas phase geometry) is not perfectly parallel to the surface. The component parallel to the surface (8.0 D) is much stronger than that perpendicular to the surface (2.6 D).

**Figure 1:**
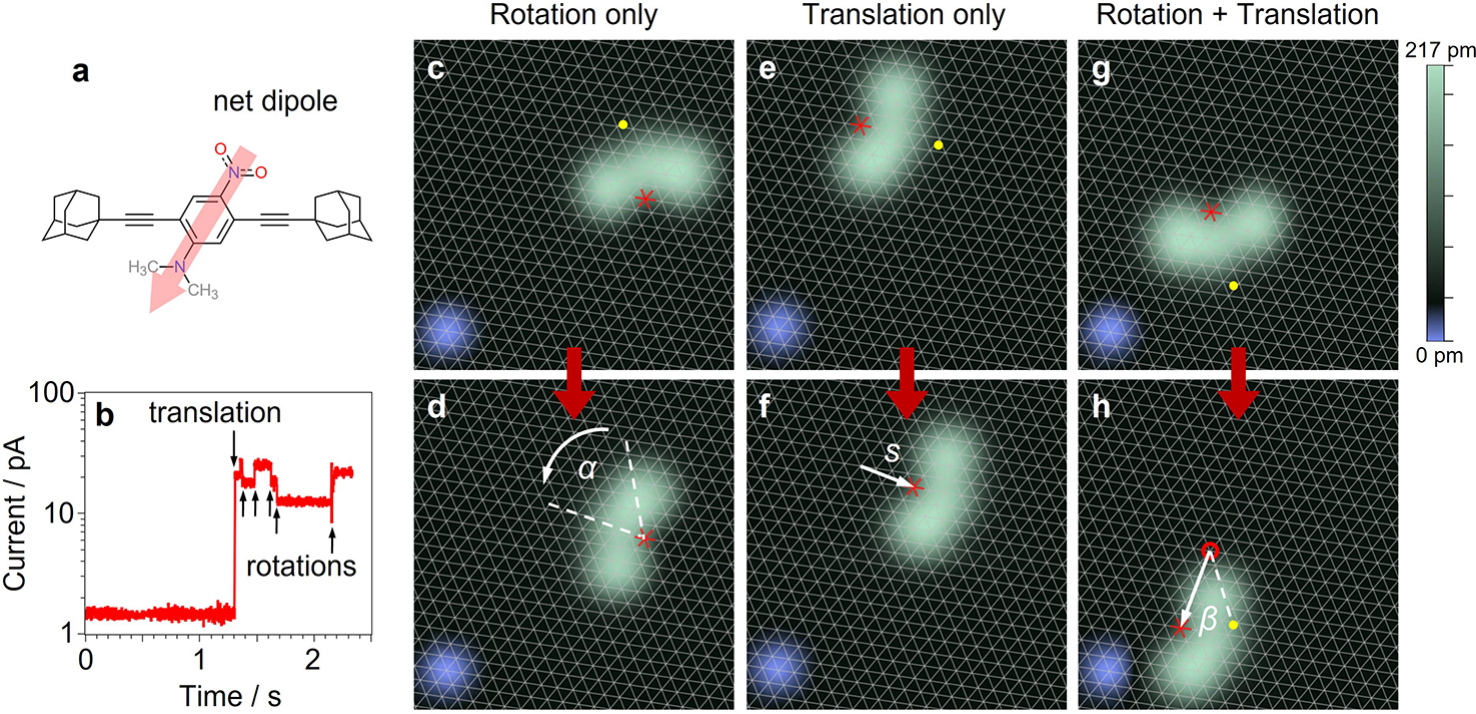
(a) Chemical structure of DDNB. (b) Typical current vs time measurement with an initial off-molecule tip position. Translation of the molecule into the position directly under the tip is recorded as a sudden jump in the current; rotations appear as smaller current fluctuations. (c–h) STM images before (top) and after (bottom) a voltage pulse of 1.8 V was applied above one and the same molecule (the CO molecule at the bottom left serves as a reference point) at the position marked with a yellow spot. Three possible outcomes are observed: (c and d) rotation only, (e and f) translation only, and (g and h) rotation and translation. Parameters α, *s*, and β indicate the rotation, displacement, and direction with respect to the pivot point (red star), respectively. The underlying copper lattice is superimposed as a grid.

Deposition of DDNB on Ag(111) can result in either isolated molecules when carried out at 80 K or close-packed honeycomb structures when done at room temperature. An STM image of an isolated molecule can be seen in Figure 1c, and its shape roughly resembles that of an unshelled peanut with the two side lobes representing each of the peripheral adamantyl groups. The molecule is known to adsorb with an O atom of the NO_2_ group positioned directly on top of a silver atom of the surface.^20^ This point acts as an anchor for the molecule around which it can pivot and rotate. Note that the molecule is chiral upon surface adsorption and is found in both enantiomeric states. Their behavior is equivalent in terms of the motions discussed below.

The rotation and hence the orientation of the molecule can be perfectly controlled via interaction of its internal dipole moment with the electric field that exists between the STM tip and the sample as has been described previously.^20^ Depending on the position of the STM tip with respect to the dipole axis as well as the polarity of the field, the molecule can be deterministically rotated into one of six equivalent orientations (in accordance with the symmetry of the underlying surface). These orientations are separated by an angle of 60°, and an example of such a rotation is shown in panels c and d of Figure 1. In this example, the molecule is first imaged using scanning parameters (0.7 V and 0.27 pA) that are known not to induce any motion (see Figure S1). Then, at the position marked in yellow, a voltage pulse of 1.8 V is performed, followed by another STM image that shows that the molecule has rotated by 60° anticlockwise (i.e., −60°) around its pivot point marked in red. From such a rotation, we can define α as the azimuthal angle through which a molecule rotates during a voltage pulse. In fact, as shown previously,^20^ such rotation can be induced at voltages greater than |±1.3 V|. At these voltages, it is common to see only rotations being induced with no shifting of the pivot point.

At higher voltages, and hence a stronger electric field in the tunnel junction, we expect that the relatively strong interaction between the nitro group and the silver surface can be overcome by the increased electrostatic force acting on the dipole and translations of the entire molecule can occur. Indeed, at voltages of at least ∼1.5 V, we find that, in addition to rotation around a fixed pivot point, translation also takes place (Figure 1e,f). Again, a voltage pulse of 1.8 V is applied in the yellow marked position in Figure 1e, and the resulting position of the molecule is shown in Figure 1f. In this example, and as indicated by the white arrow, the molecule has translated a distance, *s*, of 10.4 Å, keeping the same orientation on the surface, i.e., without a rotation. The parameter *s* is always defined as the displacement of the pivot point of the molecule (indicated with red stars in Figure 1). Note that we find the opposite behavior for negative voltage pulses (applied to the sample, while the tip is always grounded), similar to the case of the molecular rotations that result in a clockwise or anticlockwise manner for the opposite polarity,^20^ but focus on positive bias voltages below (see Figure S2).

While rotation and translation can be induced separately, translation is often coupled to rotational events, due to the fact that the electric field strength used for translation is always sufficient to induce rotation. In this case, we can see that, upon comparison of panels g and h of Figure 1, both a translation of 14 Å and a change in the rotational orientation, α, have occurred. A further parameter, β, can be defined as the angle subtended between the tip position and the final position of the pivot point with respect to the starting position of the pivot point. This can be considered the direction with respect to the tip in which the translation has occurred; for example, angles of 0° and 180° correspond to the molecule moving directly toward and directly away from the STM tip, respectively.

If the STM tip is placed for manipulation laterally displaced from the molecule (as in Figure 1g), the tunnel current measured as a function of time during the voltage pulse (Figure 1b) shows multiple features, indicating that multiple events have taken place: first, a flat, low-current signal in agreement with the fact that the molecule is not located under the tip, followed by a sudden order of magnitude jump, and, finally, many smaller jumps in the current. For voltage pulses of 1.8 V, this is the most commonly observed behavior. When such a combination of events occurs, the sequence of processes (rotation before translation or vice versa) typically cannot be identified from *I*/*t* behavior or STM images alone. In the following, we discuss how rotation and translation are related to each other and the order in which they occur.

To gain insight into the translational behavior, eight points were chosen as tip positions around the molecular contour in a clockwise direction (Figure 2a). The chemical structure shown indicates the orientation of the dipole with respect to the STM image. Despite two degenerate chiral forms of DDNB existing while adsorbed on Ag(111), only one enantiomer is used in the following analysis. At each of the eight points around the molecule, an average of 33 current versus time spectra were measured (as in Figure 1b). In addition, STM images of the molecule were taken before and after each voltage pulse to determine its orientation as well as the direction and magnitude of all translations. The three parameters described previously, *s*, β, and α, as well as the translation yield could be extracted from the data and are presented in Figure 2b–d, respectively, as radial plots at each tip position.

**Figure 2:**
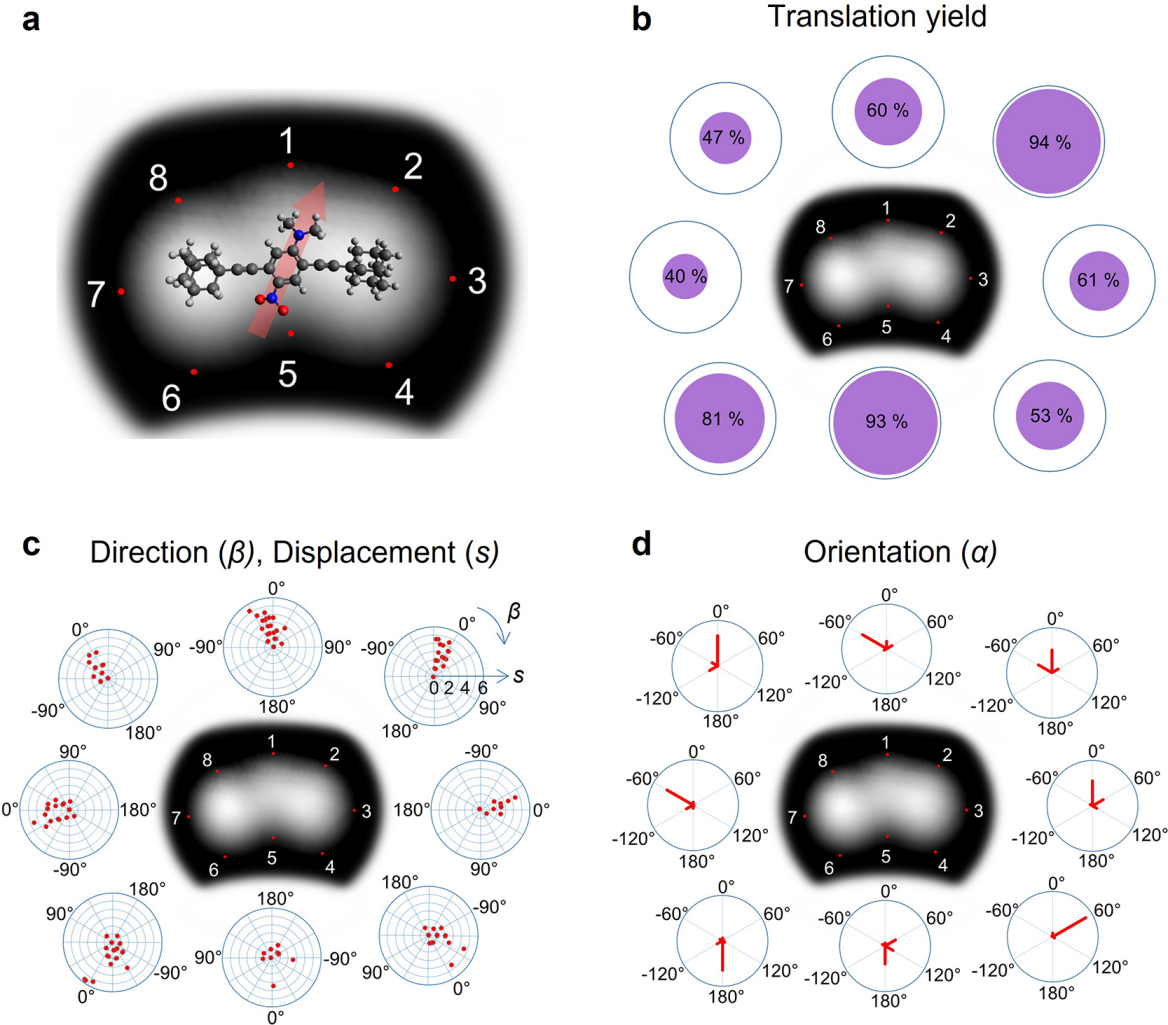
(a) STM image with the chemical structure of DDNB overlaid indicating the position of eight voltage pulse locations (the sample bias during all pulses is *V* = 1.8 V). Statistical analysis of 261 events measured at eight points around the perimeter of the molecule indicating (b) the translation yield, (c) the direction, β, and the magnitude of translation, *s*, in integer number of lattice constant, and (d) the final rotational state, α, of the molecule after the translation. The yield is defined as the number of successful manipulations, i.e., a voltage pulse followed by molecular translation.

At first glance, the orientation of the molecular dipole is directly visible in the yield map (Figure 2b) where the highest values are obtained for opposite tip positions (around 2 on one side and 5 and 6 on the other side) along the dipole moment (see Figure 2a), because the tip is closest to the molecular charge. At other positions (1, 3, 4, 7, and 8) we see significantly lower yields. This is due to the increased distance of the tip position from the dipole, and the competing forces of attraction and repulsion on the two ends of the dipole partially compensate for each other and cause an overall weaker force on the dipole.

The data in panels c and d of Figure 2 can be understood by considering three exemplary cases of a dipole in an external electric field, differing in their relative orientation of the dipole moment: parallel, antiparallel, or perpendicular (schemes a–c, respectively, in Figure 3). Because a positive bias voltage applied to the sample (as is the case for all measurements here) means that the lateral component of the field lines points in the direction of the tip, we can position the tip at the locations corresponding to these exemplary cases and hence interpret the data in Figure 2.

**Figure 3:**
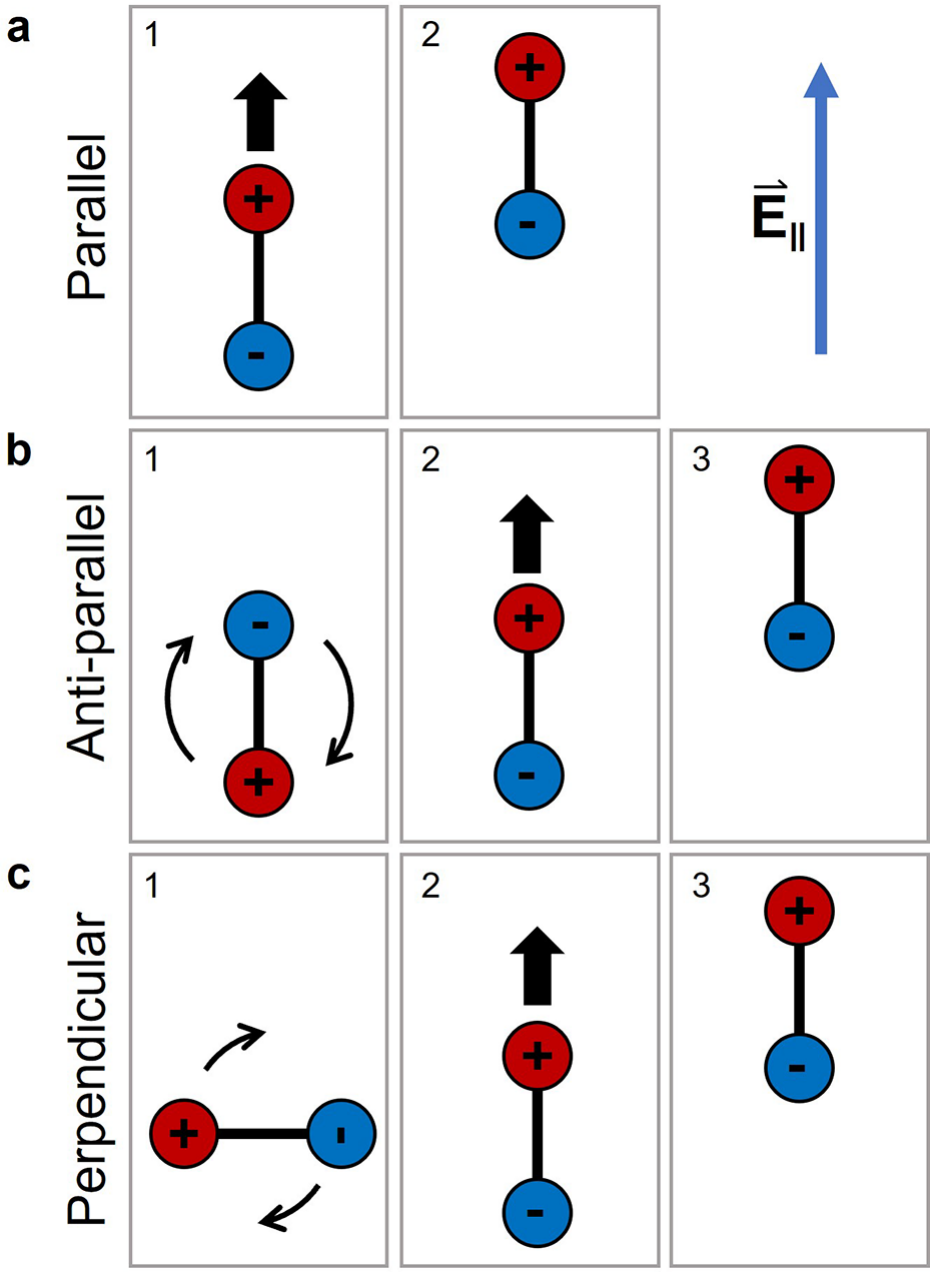
Three exemplary cases of a dipole rotating and translating in an electric field (top right). (a) Dipole moment parallel to the electric field (translation only). (b) Dipole moment antiparallel to the electric field (rotation and then translation). (c) Dipole moment perpendicular to the electric field (rotation and then translation).

*(1) Tip in Position 2 → Translation*. Translation is induced very efficiently [yield of ∼94% (Figure 2b)] with a clear direction of motion toward the STM tip (Figure 2c). In some cases, we also see rather large jumps (*s* = 5 lattice parameters, i.e., 14.4 Å) of the molecule in this direction. At the same time, the molecule preferentially maintains its orientation on the surface (maximum at α = 0° in Figure 2d) with some cases of small angle rotation (±60°). This fits exactly to scheme “parallel” (Figure 3a) in which the dipole moment is parallel with the lateral electric field component. Accordingly, the attractive interaction between the STM tip and the positively charged side of the molecular dipole prevails (i.e., with the dimethylamino group) and causes translation toward the tip with only little rotation.

*(2) Tip in Position 6 → First Rotation and Then Translation*. If the direction of the lateral electric field component is reversed with respect to the molecule, the situation changes completely. Also here, the translation is very likely (yield of ∼81%) albeit with a lower distribution of displacements (typically *s* < 3 lattice sites). However, strong rotation occurs as the molecule almost always completely changes its orientation [rotating by α = 180° (Figure 2d)]. At the same time, dislocation toward the tip (maximum around β = 0° in Figure 2c) also occurs. To understand the sequence of motion, it must be considered that the “antiparallel” configuration (Figure 3b) is present where the dipole moment is in the least favorable orientation with respect to the electric field. Hence, the molecule most likely first rotates as a translation toward the tip cannot be explained in this charge distribution. Then, after rotation to the “parallel” configuration, translation is induced (as in case 1 above) and the molecule is located closer to the tip with a flipped orientation [α = 180° as observed (Figure 2d)].

*(3) Tip in Positions 3 and 4 or 1, 7, and 8 → First Rotation and Then Translation*. The “perpendicular” scheme in Figure 3c is reached if the STM tip is placed sideways with respect to the molecular dipole. The situation is not as obvious here, but it can be seen in Figure 2d that for positions 1, 7, and 8 (i.e., at the “left” of the dipole moment) counterclockwise rotation occurs preferentially (α < 0°) while for the other side (positions 3 and 4, i.e., at the “right”) clockwise rotation prevails (α > 0°). While the −60° rotations are very dominant for positions 1 and 7, the intermediate position 8, which also shows a preference for counterclockwise rotations, exhibits a maximum at α = 0°, meaning that in most of the cases the molecule follows the tip without changing its orientation on the surface. We tentatively assign this behavior to a particular, energetically preferred, pathway in the potential energy landscape. For all of these positions (3 and 4 and 1, 7, and 8), also translation toward the tip always takes place [data scatter around β = 0° (Figure 3c)]. In this “perpendicular” configuration, the electrostatic forces should be approximately equal in size on both charges of the dipole, albeit in opposite directions. Hence, it appears to be very unlikely that translation could occur first and the only reasonable sequence is that in which the dipole moment (and consequently the molecule) is first rotated in an energetically preferred orientation (approximately approaching scheme “parallel” in Figure 3a). Accordingly, both directions of rotation are present, depending on the direction of the electric field (i.e., whether the STM tip is placed “left” or “right” of the molecule).

Then, in a second step, translation can be induced because the molecular dipole is aligned appropriately (parallel) with the electric field. Otherwise (i.e., if translation took place before rotation), competing attractive and repulsive forces on the positive and negative, respectively, poles within the molecule should result in both directions of translation at the beginning. Hence, the molecule should move not only toward the STM tip but also away from it, which is not the case (clear preference for β = 0° in Figure 2c).

*(4) Tip in Position 5*. This is a special case because the tip is located approximately above the pivot point (see Figure 1) where the molecule–surface interaction is strongest. Accordingly, there is no clear preference in the molecular orientation (Figure 2d) and various directions of motion are observed (Figure 2c). In addition, because the tip, which is on a more negative potential than the surface, is located very close to the negative charge of the molecular dipole, efficient electrostatic repulsion dominates, and a relatively high translational yield (93%) is observed.

To explore whether the surface corrugation plays a role in the observed behavior, density functional theory (DFT) calculations were performed on the DDNB/Ag(111) system. After first optimizing the structure (Figure 4a; see Methods for details), we calculated the minimum energy pathways for a single lattice site translation along the three high-symmetry directions using the nudged elastic band method (see Methods). In Figure 4a, these three directions [ (red),  (blue), and  (green)] are indicated by arrows. The corresponding calculated barriers for translation are shown in Figure 4b. We see a small, but significant, difference in the barrier for translation in the  direction of 34 meV as compared to the other two directions.

**Figure 4:**
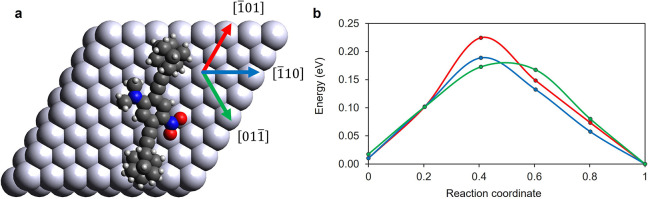
(a) DFT-optimized structure of DDNB adsorbed on Ag(111) with the three high-symmetry directions indicated by arrows. (b) Minimum energy pathways for translation of DDNB by one lattice site in each of the indicated directions. While the initial and final orientations of the molecule are constrained to be equal, the intermediate points are fully free; i.e., rotations are allowed along the pathway.

When the first atomic layer of the surface in isolation is regarded, these three surface directions are of course equivalent. However, when considering the adsorbed DDNB molecule (at the left in Figure 4a), the symmetry is broken. We can see that for the  and  directions, the molecule moves roughly perpendicular to its long axis and parallel to its dipole axis, respectively, and the barriers for translation in these directions are similar in magnitude. Along the  direction, however, the molecule is translating approximately parallel to its long axis. This effect has been reported previously^25^ and attributed to the differences in van der Waals corrugation of a molecule along equivalent close-packed surface directions.

The calculated barriers likely play a role in determining the directional preference of molecular translations that we see experimentally (despite the tip electric field not being explicitly included), and we can draw three tentative and qualitative comparisons. (1) Green is (by a small margin) the lowest barrier, and this points directly along the dipole direction in agreement with the yield diagram aligned with the dipole (Figure 2b). (2) Blue is similar in height, which would correspond to the direction along the axis joining positions 1 and 5 (Figure 2a); these also have a relatively high translational yield (Figure 2b). (3) The red direction corresponds to the highest barrier, and this is also reflected in the decreased yield in these peripheral directions (positions 4 and 8 in Figure 2b). This general agreement suggests that, while the main stimulus for translation is attraction of the dipole by the electric field, the potential energy barriers imposed by the corrugation of the Ag(111) surface also affect the translational behavior.

## Methods

*Experimental Section*. Repeated cycles of sputtering with Ar^+^ followed by annealing to ∼770 K were used to produce a clean Ag(111) surface. DDNB was deposited on the surface in the UHV chamber (base pressure of ∼1 × 10^–10^ mbar) via sublimation from a crucible held at 389 K. STM measurements were conducted at 7 K. All STM images were acquired using a commercial CreaTec instrument in the constant-current mode. The STM tip is an electrochemically etched tungsten wire. To ensure clear high-resolution imaging, the tip was subsequently sharpened through high-voltage (100 V) tip forming, which leaves a Ag tip termination.

*Computational Section*. We utilized the DFT functional PBE+D3^26,27^ together with an energy cutoff of 500 eV and standard PAW potentials^28^ for N, O, C, and H, whereas for Ag, the semicore p states have been treated as valence with VASP.^29−31^ The surface was modeled with two layers of silver atoms (with the lower layer held fixed) with a silver–silver distance of 2.83275 Å and an 8 × 8 cell, resulting in overall 128 silver atoms. To locate the transition states, a climbing-image nudged elastic band^32,33^ with four images was used, extrapolating between two translated global minima that have each been optimized on the surface.

## Supporting Information Available

Stable imaging parameters and behavior at negative biases (PDF)

## Notes

Data from this study are available from the corresponding authors upon request.
