# Supplementary material for: Directing and Understanding the Translation of a Single Molecule Dipole
Source: J Phys Chem Lett. 2023 Mar 3;14(10):2487–92. doi: 10.1021/acs.jpclett.2c03472 (PMC10026170; doi:10.1021/acs.jpclett.2c03472)
Supplement: Supplementary file 1 — jz2c03472_si_001.pdf [file jz2c03472_si_001.pdf]

Supporting Information

Directing and Understanding the Translation of a Single Molecule Dipole

Grant J. Simpson[1], Víctor García-López[2]†, A. Daniel Boese[3],

James M. Tour[2*], and Leonhard Grill[1]*

1) Department of Physical Chemistry, Institute of Chemistry, University of Graz, Heinrichstrasse 28, 8010, Graz, Austria

2) Departments of Chemistry and Materials Science and NanoEngineering, and the Smalley-Curl Institute and NanoCarbon Center, Rice University, Houston, TX, 77005, USA

3) Department of Theoretical Chemistry, Institute of Chemistry, University of Graz, Heinrichstrasse 28, 8010, Graz, Austria

* Corresponding authors: tour@rice.edu (J.M.T.) and leonhard.grill@uni-graz.at (L.G.)

S1 Stable imaging parameters

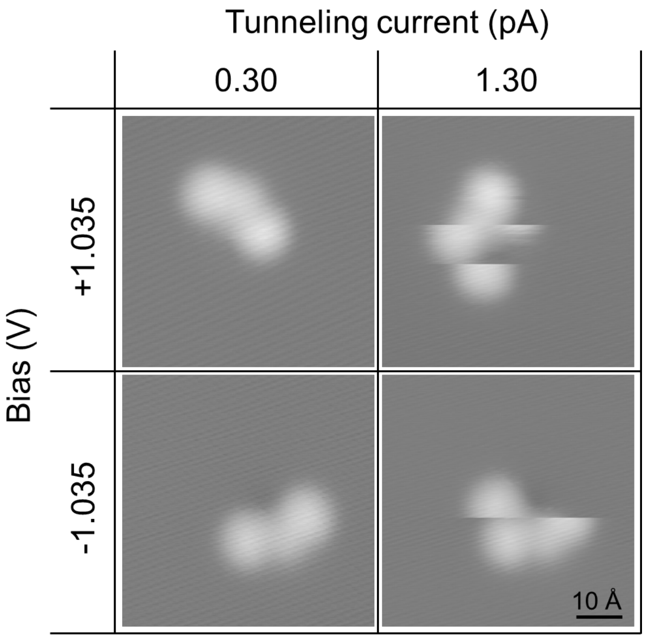

Figure S1: Four constant-current STM images demonstrating the stability of DDNB on Ag(111) under different conditions. For both bias polarities, tunneling currents of 0.30 pA (left) allow the molecule to be imaged stably but upon increasing the current to 1.30 pA (right), rapid motion during scanning is seen as abrupt discontinuities in the image.

It was found during experiments that the DDNB molecule displayed very high mobility while imaging with the scanning tunneling microscope. This was seen in images as abrupt changes in the topography which corresponded to rotations of the molecule. It was however important to understand the position and orientation both before and after a voltage pulse in order to understand how the polar molecule is affected by the field in the tunnel junction. As shown in Figure S1, we found that at both positive and negative bias polarities ($V = \pm 1.035$ V) a sub-pA current was necessary to facilitate stable imaging of the molecule. At only slightly higher values of 1.3 pA, the molecule begins rapidly rotating and at even higher values (not shown in Figure S1) the molecule is dragged by the STM tip.

S2 Behaviour at negative biases

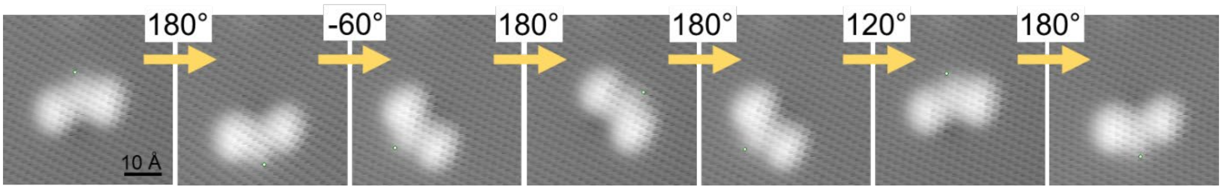

Figure S2: From left to right: a series of consecutively acquired constant-current STM images (I = 0.27 pA, V = -0.715 V) of a single DDNB molecule. After each image, a constant-height voltage pulse of -1.815 V is applied in the position marked with a dot corresponding to position 1 from the main text. The resulting angle of rotation is indicated in each case.

At negative biases, the molecule displays opposite behaviour compared to positive bias for a given STM tip position. This is exemplified in Figure S2 where voltage pulses of -1.815 V are repeatedly applied to position 1 (located at the top of the molecule in the first image of S2, also see Fig. 2a in the main text). In contrast to the case for positive bias, a strong tendency for large (120°, 180°) rotations of the molecule is seen. This is the expected behaviour due to the reversal of the electric field direction in the tunnel junction for a fixed relative orientation of the internal molecular dipole.
